# Giant gastric gastrointestinal stromal tumor with severe peritoneal dissemination controlled by imatinib therapy following debulking surgery: a case report

**DOI:** 10.1186/s13256-017-1215-5

**Published:** 2017-02-07

**Authors:** Shuichi Fukuda, Yoshinori Fujiwara, Tomoko Wakasa, Kotaro Kitani, Masanori Tsujie, Masao Yukawa, Yoshio Ohta, Masatoshi Inoue

**Affiliations:** 1 Department of Gastroenterological Surgery, Kindai University Nara Hospital, 1248-1, Otoda-cho, Ikoma, Nara 630-0293 Japan; 2 Department of Pathology, Kindai University Nara Hospital, Nara, Japan

**Keywords:** Case report, Cytoreductive surgery, Debulking surgery, Dissemination, Gastrointestinal stromal tumor, GIST, Imatinib

## Abstract

**Background:**

At the time of diagnosis, giant gastric gastrointestinal stromal tumors are sometimes associated with severe peritoneal dissemination. Unresectable gastrointestinal stromal tumors are considered a systemic disease; therefore, imatinib therapy is currently the primary treatment option in these cases.

**Case presentation:**

A 49-year-old Japanese woman was referred to our hospital with symptoms of anorexia, abdominal discomfort, and a palpable abdominal mass. Contrast-enhanced computed tomography revealed a huge mass with an irregular wall, approximately 22 cm in size, located between the posterior gastric wall and her pancreas. The tumor grew rapidly, and her abdominal symptoms worsened; therefore, a semi-urgent laparotomy was performed. The tumor had arisen from her upper stomach and was removed by wedge resection of her stomach. In addition, widely distributed multiple white nodules were noted, which were resected as far as possible. Immunohistochemical staining of the resected specimen was positive for KIT and CD34. The resected white nodules contained the same cells as the primary tumor. Based on these pathological findings, a final diagnosis of a gastric gastrointestinal stromal tumor with peritoneal dissemination was made. Imatinib was administered at 400 mg per day from 1 month postoperatively. The disease progression of the residual disseminated lesions was favorably controlled, and our patient is now doing well, 12 months after surgery.

**Conclusions:**

Imatinib therapy following debulking surgery can show dramatic effectiveness in giant gastric gastrointestinal stromal tumors with severe peritoneal dissemination.

## Background

Gastrointestinal stromal tumors (GISTs) are the most common mesenchymal tumors of the gastrointestinal tract [1]. Primary GISTs can arise anywhere along the gastrointestinal tract; however, a majority arises in the stomach (50 to 60 %) [2]. Gastric GISTs are clinically asymptomatic until they reach a significant size; therefore, a majority of GISTs are incidentally discovered either by diagnostic tests or during abdominal surgery. Giant gastric GISTs are infrequently associated with symptoms such as abdominal pain, digestive bleeding, and a palpable mass [3–5]. Such GISTs are aggressive and have the potential to cause distant metastases. Severe peritoneal dissemination is sometimes observed at the time of diagnosis of giant gastric GISTs and is associated with an extremely poor prognosis.

Recent progress in understanding the origin and molecular oncology of GISTs has contributed to a rapid improvement in their management [6]. Imatinib, which inhibits the KIT signal transduction pathway, is considered a promising therapeutic agent [7]. Unresectable GISTs are considered a systemic disease; therefore, imatinib therapy is currently the primary treatment option in these cases [8, 9]. However, in clinical practice, a precedent surgery is performed for various reasons. Surgery is beneficial to control tumor-associated complications such as abdominal pain and digestive bleeding; therefore, a precedent surgery for unresectable GISTs is sometimes necessary.

Prior to the imatinib era, surgery traditionally played only a palliative role in patients with unresectable GISTs; however, with the introduction of imatinib, it may be necessary to reassess the role of surgery in these cases. Here we report the case of a patient with a giant gastric GIST with severe peritoneal dissemination, showing favorable progression control by postoperative imatinib therapy following debulking surgery. This case demonstrated that surgery can possibly play a role even in unresectable GISTs.

## Case presentation

A 49-year-old Japanese woman was referred to our hospital with symptoms of anorexia, abdominal discomfort, and a palpable abdominal mass. She reported a 7 kg weight loss in a few months and had a medical history of hypertension and hyperlipidemia as well as a maternal history of gastric cancer. On performing a physical examination, a palpable mass was detected in her left upper abdominal quadrant. She had regular bowel movements with normal stools.

A complete blood count test revealed only mild microcytic hypochromic anemia (hemoglobin, 11.0 g/dL). A blood biochemical test revealed no abnormalities, except for an elevated lactate dehydrogenase level of 401 U/L. Her levels of tumor markers were assessed, including carcinoembryonic antigen, carbohydrate antigen 19-9, and α-fetoprotein, all of which were within normal ranges.

**Fig. 1:**
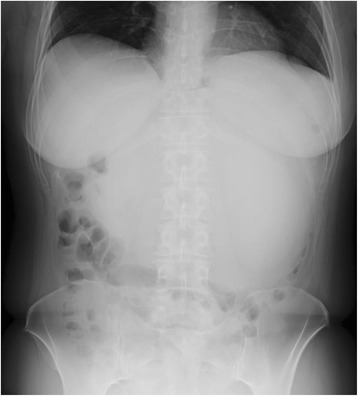
An abdominal X-ray showing a diffuse opaque area in the upper abdomen without a gastrointestinal gas shadow; this finding was accompanied by a downward dislocation of the transverse colon

**Fig. 2:**
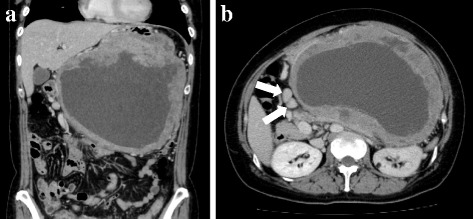
**a**, **b** Contrast-enhanced computed tomography showing a huge mass with an irregular wall, approximately 22 cm in size, located between the posterior gastric wall and the pancreas. The mass demonstrates heterogeneous contrast and internal low-density components, suggestive of necrosis. Moreover, many small nodules around the stomach (*arrows*) are observed, suggestive of enlarged lymph nodes or disseminated lesions

An abdominal X-ray revealed a diffuse opaque area in her upper abdomen without a gastrointestinal gas shadow; accompanying the finding, her transverse colon was dislocated downward (Fig. 1). Endoscopy showed compression of the posterior wall of her gastric body along with mucosal redness. Several biopsy specimens were taken, which showed evidence of erosive atrophic gastritis. Contrast-enhanced computed tomography (CT) revealed a huge mass with an irregular wall, approximately 22 cm in size, located between the posterior gastric wall and her pancreas (Fig. 2). The mass demonstrated heterogeneous contrast and internal low-density components, suggestive of necrosis. The mass was mainly supplied by her left gastric artery branches. Moreover, many small nodules around her stomach were observed, suggestive of enlarged lymph nodes or disseminated lesions. There were no signs of hepatic masses. Based on these findings, a mesenchymal tumor, such as a GIST, leiomyoma, leiomyosarcoma, or a neurogenic tumor, was suspected.

The tumor grew rapidly, and her abdominal symptoms worsened; therefore, a semi-urgent laparotomy was performed, although a definitive diagnosis could not be preoperatively made. After gastrocolic ligament division, a giant extraluminal tumor arising from the posterior wall of her upper stomach on the lesser curvature was noted. Furthermore, multiple white nodules, suspected of being disseminated lesions, were widely distributed in her greater omentum, lesser omentum, transverse mesocolon, and retroperitoneum. Serous ascites was also seen in the pouch of Douglas. The giant tumor adhered to her pancreas, transverse mesocolon, and transverse colon; however, the tumor was removed by wedge resection of her stomach following ligation of the vessels supplying the mass, without resecting other organs. The radical resection of the multiple white nodules was impossible; therefore, the nodules were resected as far as possible.

**Fig. 3:**
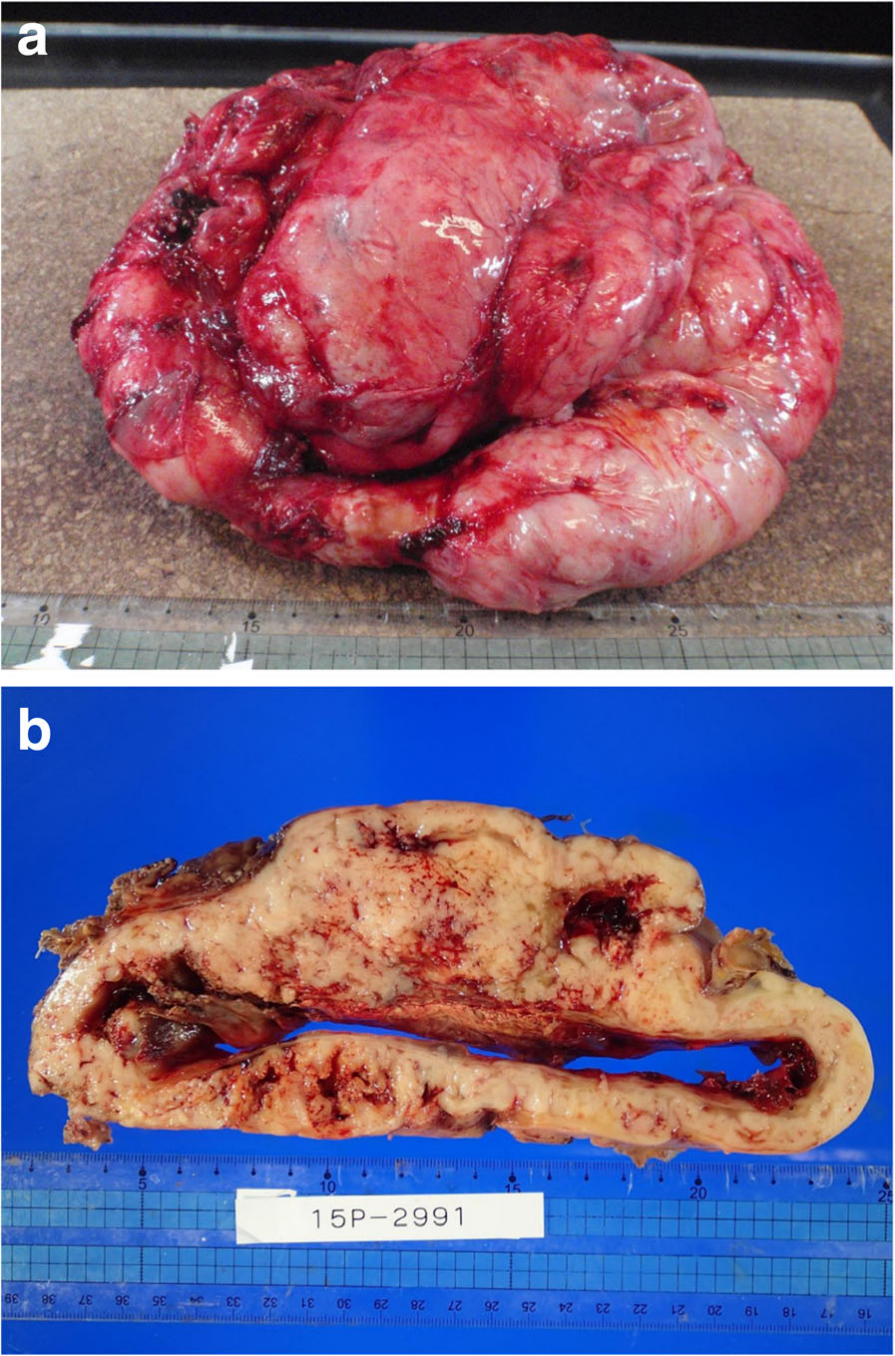
**a** The resected specimen is a well-circumscribed tumor. **b** The cut surface of the tumor reveals a white–gray solid mass with coagulative necrosis, accompanying a large central cavity

The resected specimen was a well-circumscribed tumor (Fig. 3a). The cut surface of the tumor revealed a white–gray solid mass with coagulative necrosis, accompanying a large central cavity (Fig. 3b).

**Fig. 4:**
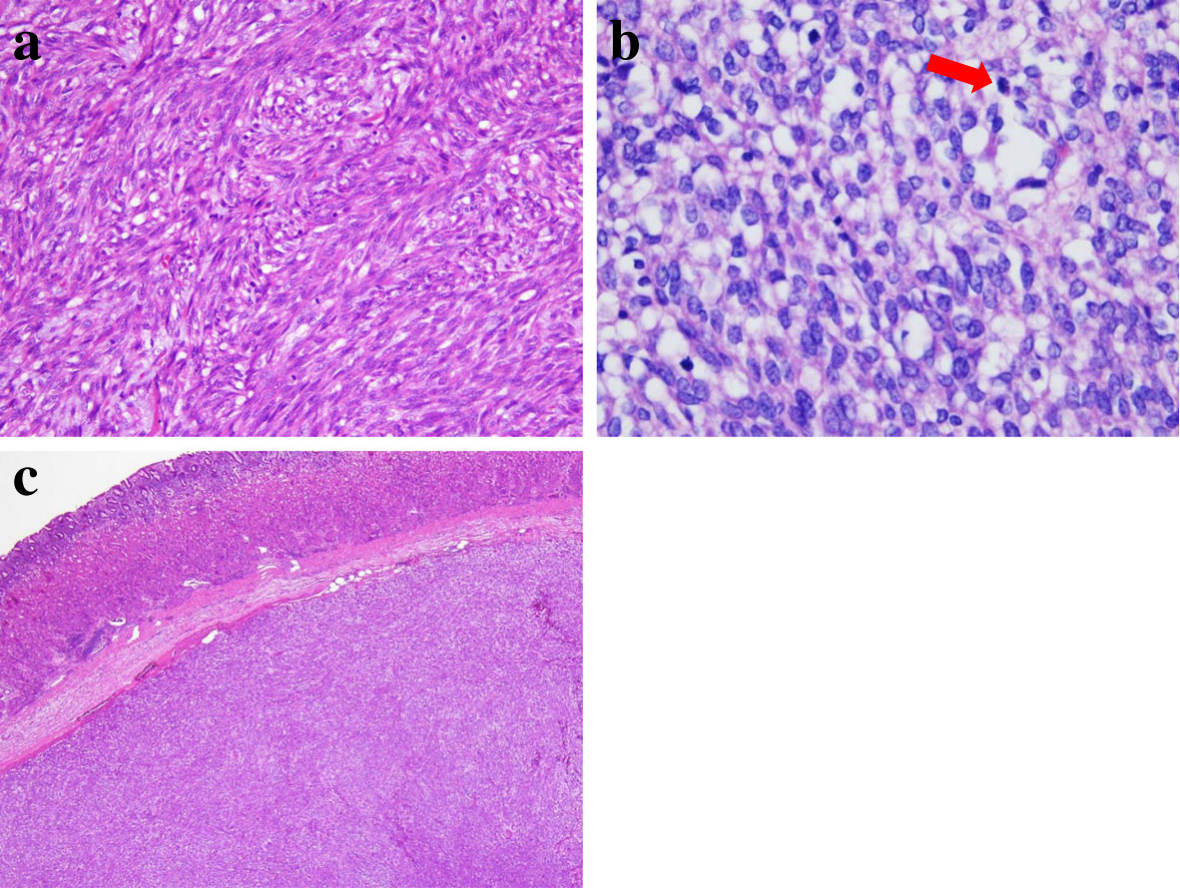
**a** The microscopic findings of hematoxylin–eosin staining showing a bundle-like growth of spindle-shaped tumor cells. **b** Nuclear atypia is prominent, and the mitotic count is over 150 per 50 high-power fields. The *red arrow* shows mitosis. **c** Tumor cells growing externally from the proper muscle layer of the stomach

**Fig. 5:**
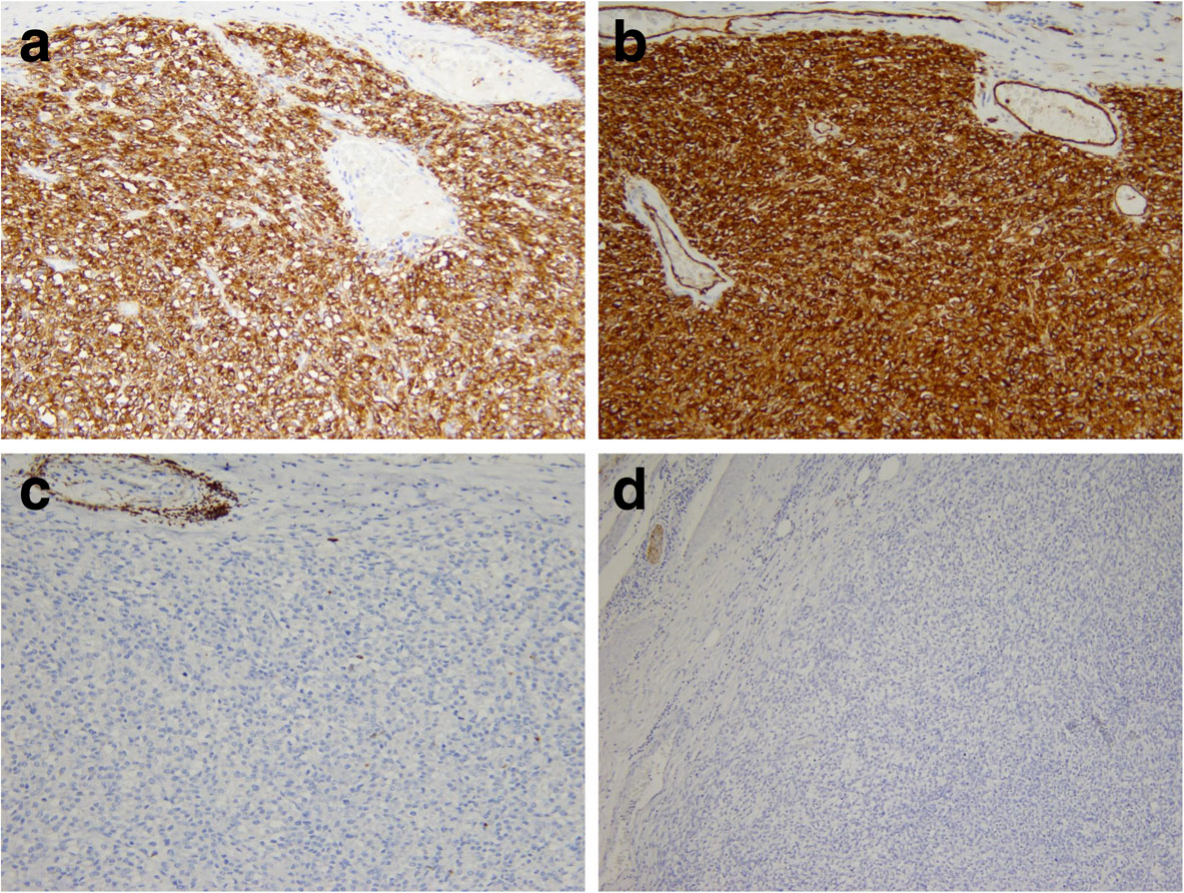
Immunohistochemical staining showing that the tumor is positive for KIT (**a**) and CD34 (**b**) and negative for desmin (**c**) and S-100 protein (**d**)

**Fig. 6:**
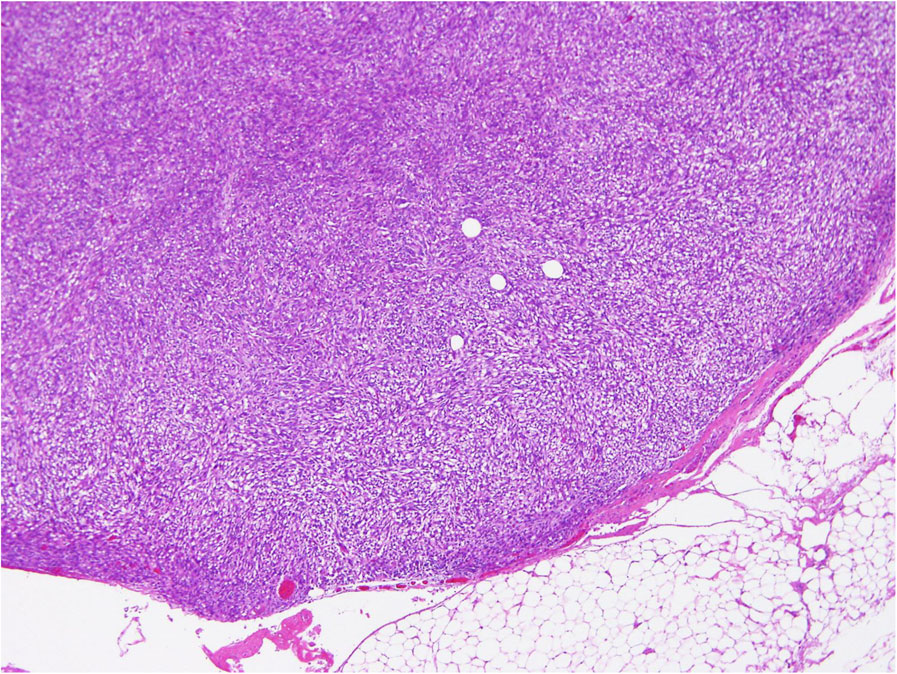
The resected white nodules at the greater omentum contain the same spindle-shaped cells same as the primary tumor

The microscopic findings of hematoxylin–eosin staining revealed a bundle-like growth of spindle-shaped tumor cells, with acidophilic cytoplasm and enlarged nuclei accompanying increased chromatin levels (Fig. 4a). Nuclear atypia was prominent, and the mitotic count was over 150 per 50 high-power fields (Fig. 4b). Tumor cells grew externally from the proper muscle layer of her stomach (Fig. 4c). The resection margins were free of tumor cells. Immunohistochemical staining revealed that the tumor was positive for KIT and CD34 and negative for desmin and S-100 protein (Fig. 5). The MIB-1 labeling index of the tumor cells was 40 to 50 %. On histologic examination, the resected white nodules had the same appearance as the primary tumor and contained no components of lymph nodes (Fig. 6). Based on these pathological findings, a final diagnosis of gastric GIST with peritoneal dissemination was made. According to the modified Fletcher’s classification, our patient was classified in the high-risk category [10].

**Fig. 7:**
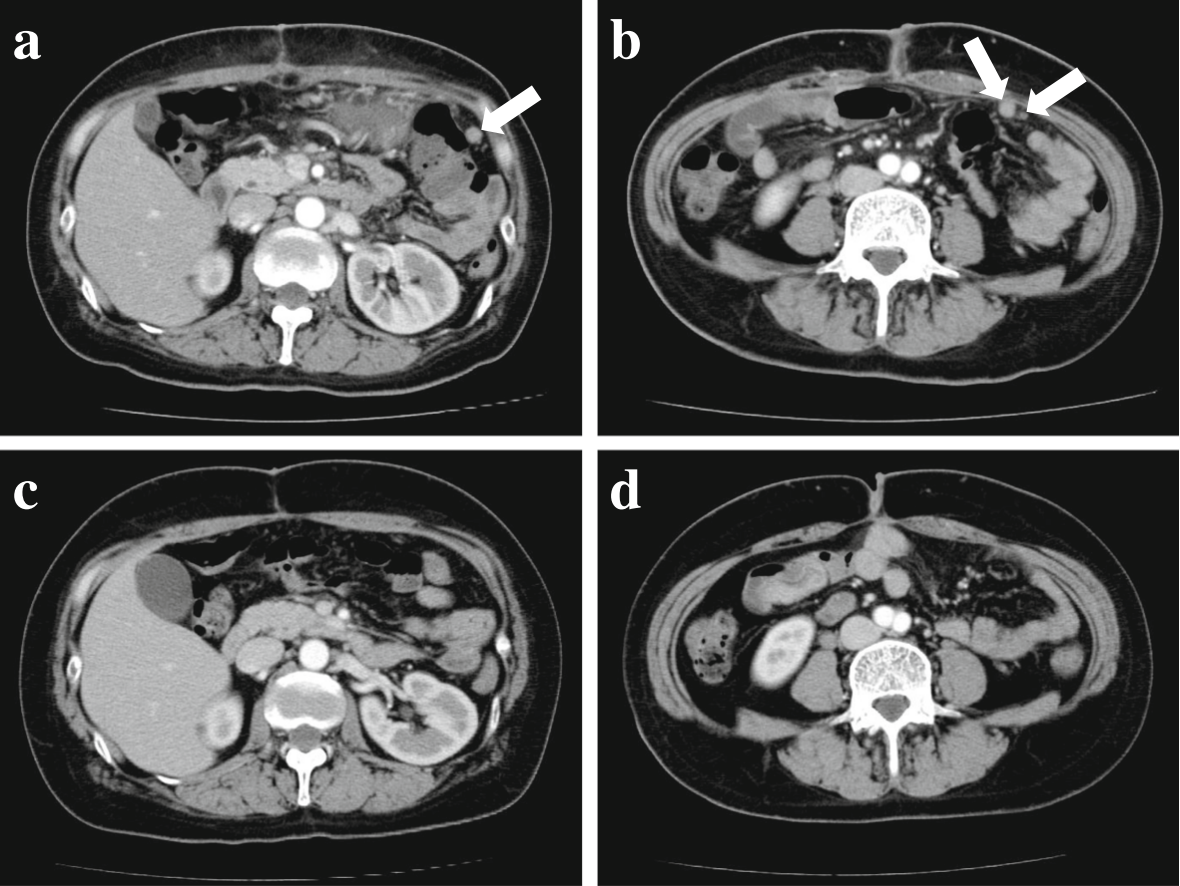
**a**, **b** Postoperative computed tomography, before imatinib administration, showing several recognizable disseminated lesions in the abdominal cavity (*arrows*). **c**, **d** Follow-up computed tomography, 9 months after imatinib administration, showing ambiguous residual disseminated lesions

Our patient had an uneventful postoperative course and was discharged from our hospital on postoperative day 15. Postoperative CT showed several recognizable disseminated lesions in her abdominal cavity (Fig. 7a, b). Treatment with imatinib at 400 mg per day was started from 1 month postoperatively. The treatment was well tolerated, with grade 1 eyelid swelling and grade 1 skin rash, which improved immediately. A follow-up CT, 9 months after imatinib administration, revealed ambiguous residual disseminated lesions (Fig. 7c, d). She is now doing well, 12 months after surgery. Imatinib administration at 400 mg per day is to be continued until disease progression is favorably controlled, with follow-up CT planned once every 3 months.

## Discussion

Complete tumor resection with negative margins is the principle treatment for resectable GISTs, whereas imatinib therapy is the principle treatment for unresectable GISTs [11, 12]. Recent studies have demonstrated that the prognosis of unresectable GISTs can be improved by surgery following their conversion to resectable tumors by imatinib therapy [13, 14]. Du *et al*. reported that the 2-year progression-free survival in patients with advanced GISTs who underwent surgery for residual lesions after having responded to imatinib therapy was higher than that in patients with advanced GISTs who received imatinib therapy alone (88.4 % versus 57.7 %) [13]. Furthermore, Rubió-Casadevall *et al*. reported that the median survival in patients with advanced GISTs who underwent surgery for residual lesions after having responded to imatinib therapy was longer than that in patients with advanced GISTs who received imatinib therapy alone (87.6 months versus 59.9 months) [14]. However, the effectiveness of imatinib therapy following a precedent surgery for unresectable GISTs remains unclear. For controlling tumor-associated complications, we performed a precedent surgery that was non-radical and subsequently administered postoperative imatinib therapy for the residual lesions. As a consequence of administering imatinib therapy following debulking surgery, the disease progression of the residual disseminated lesions was favorably controlled. The present case suggests that this treatment strategy for giant gastric GISTs with severe peritoneal dissemination can show dramatic effectiveness.

In our case, a precedent surgery was performed prior to imatinib administration for an unresectable giant GIST. As previously reported, serious adverse events, such as gastrointestinal or intra-abdominal bleeding due to tumor degeneration, occur in approximately 5 % of giant GISTs treated with imatinib [7]. Therefore, the application of imatinib must be carefully considered, particularly for giant GISTs. Furthermore, it is commonly assumed that GISTs with severe peritoneal dissemination showing conversion to resectable tumors by imatinib therapy are relatively rare. Thus, we propose that a precedent surgery followed by imatinib therapy is an attractive treatment option for treating giant GISTs with severe peritoneal dissemination. As previously reported, the development of secondary resistant lesions was related to the amount of residual tumor, which is consistent with the hypothesis that a larger number of tumor cells are quantitatively proportional to a greater likelihood of harboring more resistant clones [15, 16]. Therefore, tumor debulking, as far as possible, is desirable for delaying secondary resistance to imatinib.

Our case demonstrated that surgery can possibly play a role even in unresectable GISTs. Prior to the introduction of imatinib, outcomes for patients with unresectable GISTs were exceedingly poor, with median survival times ranging from 10 to 20 months and a 5-year survival of <10 % [17, 18]. With the introduction of tyrosine kinase inhibitors, including imatinib, sunitinib, and regorafenib, the prognosis for unresectable GISTs has tremendously improved, and the treatment strategy for these GISTs has considerably changed [7, 19, 20]. A combination of surgery and tyrosine kinase inhibitor therapy may be a promising strategy, with a large role for surgery in unresectable GISTs in addition to resectable GISTs.

At present, 11 months following imatinib administration, the disease progression of the severe peritoneal dissemination in our patient has been favorably controlled. However, the appearance of resistant lesions may become a problem later as the median progression-free survival has been shown to be approximately 2 years for patients treated with imatinib [6, 12]. For the early detection of disease progression, frequent follow-up CT at 3-month intervals has been planned [21]. Imatinib has been planned to be continuously administered as its interruption may result in rapid disease progression [22].

As this was a single-patient case report, these findings need to be confirmed by the accumulation of prospective evidence from more patients in multiple institutions. Gastric GISTs are relatively rare; therefore, the number of patients treated in a single institution is limited. However, the current findings provide important information that can contribute to the development of a treatment strategy for giant gastric GISTs with severe peritoneal dissemination.

## Conclusions

We reported the case of a patient with a giant gastric GIST with severe peritoneal dissemination showing favorable progression control by imatinib therapy following debulking surgery. The present case suggests that imatinib therapy following debulking surgery for giant gastric GISTs with severe peritoneal dissemination can show dramatic effectiveness.

## Abbreviations

CT: Computed tomography
GIST: Gastrointestinal stromal tumor
